# Epithelioid hemangioendotheliomas with *TFE3* gene translocations are compossible with *CAMTA1* gene rearrangements

**DOI:** 10.18632/oncotarget.7060

**Published:** 2016-01-28

**Authors:** Seok Joo Lee, Woo Ick Yang, Woo-Suk Chung, Sang Kyum Kim

**Affiliations:** ^1^ Department of Pathology, Yonsei University Medical Center, Seoul, South Korea; ^2^ Department of Diagnostic Radiology, Konyang University Hospital, Daejeon, South Korea

**Keywords:** epithelioid hemangioendothelioma, TFE3, YAP1, CAMTA1, WWTR1, Pathology Section

## Abstract

Epithelioid hemangioendotheliomas (EHEs) are vascular tumors of intermediate malignancy that can undergo high-grade malignant transformations. EHEs have been characterized by tumor-specific WW domain-containing transcription regulator 1(*WWTR1*)-calmodulin-binding transcription activator 1 (*CAMTA1*) translocations, and recently, a novel Yes-associated protein 1 (*YAP1*)-transcription factor E3 (*TFE3*) gene fusion was identified in EHEs. In this study, we examined the expression levels of TFE3 and CAMTA1 via immunohistochemical staining and identified chromosomal alterations using fluorescence *in situ* hybridization (FISH) assays and RT-PCR tests. Although all of the EHEs were CAMTA1-positive in immunohistochemical staining, only five out of 18 EHEs (27.78%) positively expressed nuclear TFE3. The five TFE3-positive EHEs exhibited *TFE3* gene break-apart in FISH assays. *YAP1-TFE3* gene fusions were confirmed by RT-PCR. Interestingly, we observed *CAMTA1* gene break-apart in all of the five TFE3-positive EHEs via FISH assays, and four out of the five TFE3-positive EHEs exhibited *WWTR1-CAMTA1* gene fusions via RT-PCR. These results indicate that these two chromosomal alterations are not mutually exclusive but compossible in EHEs. Finally, primary tumor sites in TFE3-positive EHEs consistently contained single masses (*P* = 0.0359) with larger sizes (*P* = 0.0550) compared to TFE3-negative EHEs. Similar to previous reports, we observed well-formed vessels more frequently in TFE3-positive EHEs than in TFE3-negative EHEs (*P* = 0.0441). In addition, TFE3-positive EHEs tended to more frequently demonstrate high-grade nuclear atypia (*P* = 0.0654) and hypercellularity (*P*=0.0987) than TFE3-negative EHEs. Thus, we have now established two clinically distinct subgroups of EHEs: TFE3-positive and TFE3-negative EHEs.

## INTRODUCTION

Epithelioid hemangioendotheliomas (EHEs) are malignant angiocentric vascular neoplasms composed of chains and cords of epithelioid endothelial cells distributed in a myxohyaline stroma [1]. EHEs affect patients of all ages and arise in virtually any body site. High-risk patients with large tumor sizes and frequent mitosis have a poorer disease-specific survival of 59% compared to 100% survival of patients whose tumors lacked these features [2].

Several studies have reported an EHE-specific translocation resulting in the fusion of the WW domain-containing transcription regulator 1 (*WWTR1*) gene on 3q23-24 with the calmodulin-binding transcription activator 1 (*CAMTA1*) gene on 1p36 [3-5]. Recently, researchers identified a novel fusion between the Yes-associated protein 1 (*YAP1*) gene and the transcription factor E3 (*TFE3*) gene, which defined a distinct subset of EHEs characterized by somewhat different histological morphologies, including focally well-formed vascular channels and variably solid architectures, clinically occurred in young adults [6, 7].

However, it remains unclear if these two distinct chromosomal alterations, the *WWTR1-CAMTA1* fusion and the *YAP1-TFE3* fusion specifically, are mutually exclusive in EHEs. In this study, we assess *TFE3* rearrangements in EHEs to verify the newly proposed classification system for EHEs and examine the details of the clinicopathological characteristics of *TFE3* rearranged EHEs.

## RESULTS

### Clinicopathologic data of EHEs

A total of 18 patients with EHEs (eight men and ten women) were included in the study, and the age at diagnosis ranged between 23-82 years (mean ± standard deviation: 49.89 ± 15.26) (Table 1). Tumors of various sizes (4.85 ± 4.77 cm) arose from a variety of organs. Eight out of 18 EHEs (44.4%) occurred in the liver, and tumors most frequently spread to the lungs (7/8, 87.5%). At diagnosis, eight out of 18 EHEs (44.4%) presented as multiple nodules in the primary lesion. Two out of 18 patients of EHE had tumor recurrence (11.1%) during follow-up periods (78.11 ± 16.36 months).

**Table 1:** Clinical presentation of EHEs

Case	Sex/Age	Primary site	Tumor size^a^ (cm)	Multiplicity^b^	Metastatic site	Treatment	Recurrence^c^	Survival (follow-up month)
1	M/59	Tongue	-^d^	Single	-	STx	No	Alive (241)
2	M/26	Liver	1.0	Multiple	Lung	STx, CTx	No	Alive (240)
3	M/46	Inguinal area	2.0	Multiple	Lung, lymph node, brain, breast	STx	No	Dead (7)
4	M/42	Liver	10.7	Single	Lung	STx, TACE	No	Alive (145)
5	M/46	Liver	1.6	Multiple	-	STx	No	Alive (105)
6	F/39	Lung	2.8	Single	-	STx	No	Alive (95)
7	F/54	Thigh	19.0	Single	-	STx, RTx	No	Alive (83)
8	F/57	Axilla	1.5	Single	-	STx	No	Alive (75)
9	F/82	Liver	10.8	Multiple	-	STx, TACE	No	Alive (73)
10	F/37	Liver	4.2	Multiple	Lung	STx, CTx	No	Alive (67)
11	F/35	Liver	3.3	Multiple	-	STx	No	Alive (59)
12	F/55	Upper arm	1.9	Single	Lung	STx, CTx	Yes	Alive (58)
13	M/51	Liver	4.0	Multiple	-	STx	No	Alive (42)
14	F/23	Submandible	1.5	Single	-	STx	No	Alive (40)
15	M/70	Lung	6.0	Single	Lymph node, liver, adrenal gland	STx, RTx	No	Dead (2)
16	F/69	Parapharynx	3.8	Single	-	STx	Yes	Alive (33)
17	M/48	Femur	7.2	Single	Stomach, lung	STx, CTx, RTx	No	Alive (32)
18	F/59	Liver	1.2	Multiple	Lung	STx	No	Alive (6)

a Measured the longest diameter of the largest mass when the tumor presented as multiple nodules.

b Indicates whether single or multiple lesions were present in the primary tumor site at diagnosis.

c Tumor recurrence observed over a follow-up period of two years.

d Value could not be determined because an imaging study or mass excision had not been performed.

M: male, F: female.

STx: surgical resection, CTx: chemotherapy, RTx: radiation therapy, TACE: transarterial chemoembolization.

**Figure 1:**
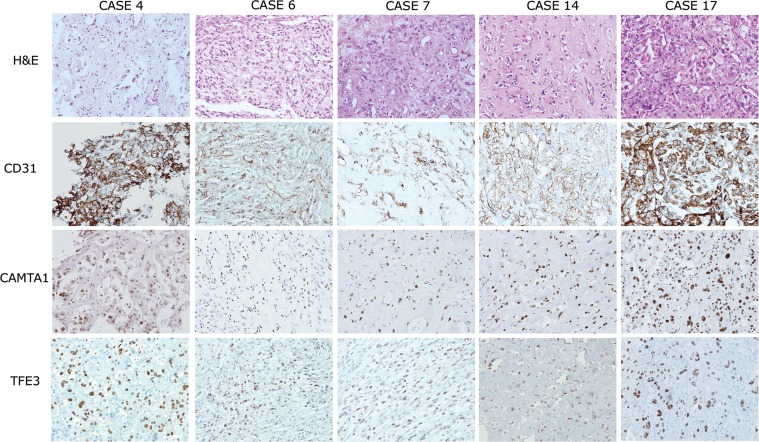
Histological features and immunohistochemical expression profiles of TFE3-positive EHEs EHEs composed of vasoformative CD31-positive epithelioid tumor cells in a myxohyalinestroma (upper two lanes). Five out of 18 EHEs demonstrated strong simultaneous nuclear expression of CAMTA1 and TFE3 (lower two lanes, respectively). 200× magnification.

Upon microscopic examination, many cases were composed of chains and cords of epithelioid tumor cells distributed in a myxohyaline stroma (Figure 1, Supplementary Data 1). All of the tumors showed strong expression of CD31 or CD34 via immunohistochemistry, indicating that they were vascular tumors. Taken together, the diagnoses of these cases were consistent with EHE.

Next, we performed immunohistochemical staining of CAMTA1 and TFE3 (Figure 1 and Table 2). As a result, all of the EHEs demonstrated strong nuclear staining of CAMTA1. Additionally, TFE3 nuclear expression in tumor cells was identified in five out of 18 cases (27.8%). As previously described [6, 7, 11], we also found well-formed vascular channels in the five TFE3-positive EHEs on a microscopic examination. Because CAMTA1 expressions were also observed in the five TFE3-positive EHEs at the same time, we decided that other tests were necessary to determine expression status of CAMTA1 and TFE3 in EHEs.

**Table 2:** Histologic findings and immunohistochemical staining results of EHEs

Case	Histologic feature	CAMTA1 expression	TFE3 expression
1	Spindle cell feature, foamy cytoplasm	+	−
2	Abundant stroma	+	−
3	Abundant stroma	+	−
4	Abundant stroma	+	+
5	Abundant stroma	+	−
6	Well-formed blood vessels, spindle cell feature, foamy cytoplasm, mitotic activity (2/10 HPFs), hypercellularity	+	+
7	Well-formed blood vessels, spindle cell feature, abundant stroma, focal high-grade nuclear atypia, hypercellularity	+	+
8	Well-formed blood vessels, mitotic activity (1/10 HPFs), hypercellularity	+	−
9	Abundant stroma, mitotic activity (1/10 HPFs), tumor necrosis	+	−
10	Abundant stroma, tumor necrosis	+	−
11	Abundant stroma	+	−
12	Spindle cell feature, abundant stroma, mitotic activity (1/10HPFs)	+	−
13	Abundant stroma, mitotic activity (1/10 HPFs), tumor necrosis	+	−
14	Abundant stroma, mitotic activity (1/10 HPFs), tumor necrosis	+	+
15	Spindle cell feature, abundant stroma, foamy cytoplasm	+	−
16	Spindle cell feature, foamy cytoplasm, mitotic activity (1/10 HPFs)	+	−
17	Abundant stroma, moderate to focal high-grade nuclear atypia, foamy cytoplasm, mitotic activity (1/10 HPFs), tumor necrosis	+	+
18	Abundant stroma	+	−
Total		18/18 (100.0%)	5/18 (27.8%)

+: positive, −: negative

### *YAP1-TFE3* gene fusions are compatible with *WWTR1-CAMTA1* translocations in EHEs

**Figure 2:**
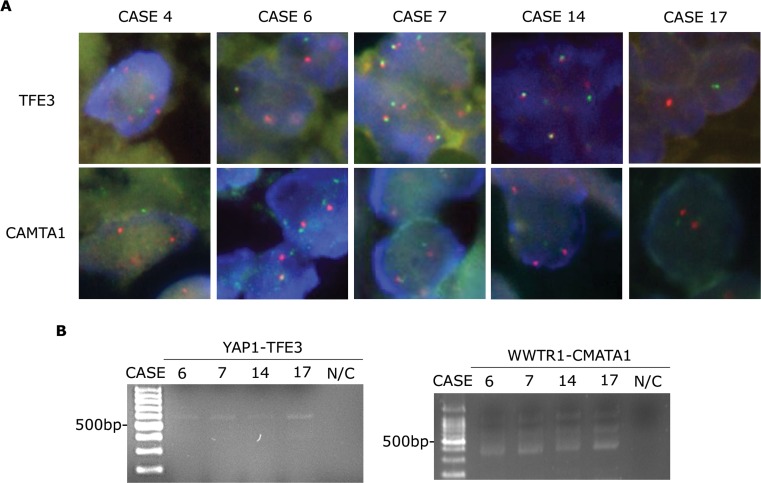
*CAMTA1* translocation in EHEs with *TFE3* rearrangements **A.** FISH assay results for *TFE3* and *CAMTA1* break-apart showing separated green and orange signals (1,000× magnification). **B.** RT-PCR assay results identifying *YAP1-TFE3* (556 bp, left panel) and *WWTR1-CAMTA1* fusion transcripts (400 bp, right panel). N/C, negative control.

To verify *TFE3* gene status, we performed FISH assays using TFE3 break-apart probes. We confirmed the presence of *TFE3* gene translocations in the five EHEs that were immunohistochemically TFE3-positive (Figure 2A, upper panels). To ensure the presence of *YAP1-TFE3* gene fusions, we performed RT-PCR using a *YAP1* exon 1 forward primer and a *TFE3* exon 6 reverse primer. *YAP1-TFE3* fusion transcripts were identified in four out of five TFE3-positive cases (Figure 2B and Table 3).

**Table 3:** Chromosomal alteration of *TFE3* and *CAMTA1*

Case	*TFE3* status		*CAMTA1* status	
	FISH^a^	RT-PCR^b^	FISH^c^	RT-PCR^d^
4	+	Unknown^e^	+	Unknown^e^
6	+	+	+	+
7	+	+	+	+
14	+	+	+	+
17	+	+	+	+

a Interpreted as positive (+) when at least 10 out of 50 tumor cells (20%) showed separate green and orange signals using *TFE3* break-apart probes.

b Interpreted as positive (+) when the *YAP1-TFE3* fusion transcript was observed via RT-PCR.

c Interpreted as positive (+) when at least 10 out of 50 tumor cells (20%) showed separate green and orange signals using *CAMTA1* break-apart probes.

d Interpreted as positive (+) when the *WWTR1-CAMTA1* fusion transcript was observed via RT-PCR.

e Value could not be determined because RNA extraction was failed.

In parallel with the *YAP1-TFE3* gene fusion assessments, we examined *CAMTA1* gene status. As before, we performed FISH assays using CAMTA1 break-apart probes in five EHEs that were immunohistochemically TFE3-positive (Figure 2A, lower panels). Unlike previous reports which claimed mutually exclusive occurrence of *WWTR1-CAMTA1* translocation and *YAP1-TFE3* gene fusion [6, 7, 11], tumor cells from the TFE3-positive EHEs showed separate signals, indicating the presence of *CAMTA1* translocations. We performed RT-PCR using a *WWTR1* exon 4 forward primer and a *CAMTA1* exon 9 reverse primer, and, interestingly, four TFE3-positive EHEs produced *WWTR1-CAMTA1* fusion transcripts (Figure 2B and Table 3). Unfortunately, we were unable to extract RNA in one last case; thus, we could not evaluate the presence of *YAP1-TFE3* or *WWTR1-CAMTA1* fusions via RT-PCR tests in this case.

Next, we examined the 13 EHEs that were immunohistochemically TFE3 negative. FISH revealed that break apart occurred only within the *CAMTA1* gene but not the TFE3 gene in these patients (Supplementary Data 2A). We repeatedly attempted to extract mRNA from these patients to perform RT-PCR but failed in 7 of 13 TFE3-negative EHE cases presumably due to mRNA degradation of the formalin-fixed, paraffin-embedded samples. RT-PCR of the other six cases from which we successfully extracted mRNA supported the presence of fusion transcripts of *WWTR1-CAMTA1* genes but not *YAP1-TFE3* genes (Supplementary Data 2B).

### Comparison of the clinical and histologic presentation of TFE3-positive and TFE3-negative EHEs

Using immunohistochemistry, the clinical features of five TFE3-positive EHEs were compared to those of 13 TFE3-negative EHEs (Table 4). At diagnosis, patients with TFE3-positive EHEs tended to be younger (41.20 ± 5.30 years) than patients with TFE3-negative EHEs (53.23 ± 4.30 years, *P* = 0.1383). Additionally, the longest diameter of single tumor size was much larger in TFE3-positive EHEs (8.24 ± 3.14 cm) compared with TFE3-negative EHEs (3.44 ± 0.80 cm, *P* = 0.0550). Although TFE3-negative EHEs demonstrated small tumor sizes, they were more likely to present multiple lesions in the primary tumor site whereas TFE3-positive EHEs exhibited a single lesion (*P* = 0.0359).

**Table 4:** Comparison of the clinical presentations of TFE3-positive and TFE3-negative EHEs

(%)	TFE3 (+) EHE	TFE3 (−) EHE	*P*-value
Case number	5	13	
Age at diagnosis (year)	41.20 ± 5.23	53.23 ± 4.30	0.1383
Sex (Male:Female)	2:3	6:7	1.0000
Follow-up period (month)	79.00 ± 20.44	77.77 ± 37.66	0.9743
Overall survival	5/5 (100.0)	11/13 (84.6)	0.3598^a^
Tumor size (cm)	8.24 ± 3.14	3.44 ± 0.80	0.0550
Multiplicity	0/5 (0.0)	8/13 (61.5)	0.0359
Local Recurrence ^b^	0/5 (0.0)	2/13 (15.4)	1.0000
Distant Metastasis	2/5 (40.0)	6/13 (46.2)	1.0000

a A statistical difference of overall survival between patients with TFE3-positive and TFE3-negative EHEs by the Kaplan-Meier analysis

b Tumor recurrence in EHE patients was observed over a follow-up period of two years.

In the TFE3-positive group, no local recurrence (0.0%, *n* = 0/5) occurred, but two distant metastases (40.0%, *n* = 2/5) developed. Meanwhile, in the TFE3-negative group, two local recurrences (15.4%, *n* = 2/13), and six distant metastases (53.9%, *n* = 6/13) developed. No statistically significant difference was observed in the rate of local recurrence (*P* = 1.0) and distant metastatic (*P* = 1.0) between TFE3-positive and TFE3-negative EHEs (Table 4). When we compared the overall survival between patients with TFE3-positive and TFE3-negative EHEs by Kaplan-Meier analysis, there was no statistically significant difference (Supplementary Data 3; *P* = 0.3598).

We compared the histologic features of EHE cases according to their TFE3 expression state but observed varying degrees of myxohyalinized stroma, tumor necrosis, mitotic activity, spindle and epithelioid cytologic features, and foamy and eosinophilic cytoplasm that seemed irrelevant to their TFE3 expression (*P* > 0.05, Table 5 and Supplementary Data 1). Similar to previous reports [6, 7], we observed well-formed vessels more frequently in TFE3-positive EHEs than in TFE3-negative EHEs (*P* = 0.0441). In addition, TFE3-positive EHEs tended to more frequently demonstrate high-grade nuclear atypia (*P* = 0.0654) and hypercellularity (*P* = 0.0987) than TFE3-negative EHEs, although the difference did not reach statistical significance.

**Table 5:** Comparison of the histologic features of TFE3-positive and TFE3-negative EHEs

(%)	TFE3 (+) EHE	TFE3 (−) EHE	*P*-value
Case number	5	13	
Presence of spindle cytologic feature	2/5 (40.0)	4/13 (30.8)	1.0000
Presence of foamy/feathery cytoplasm	2/5 (40.0)	4/13 (30.8)	1.0000
Presence of high-grade nuclear atypia	2/5 (40.0)	0/13 (0.0)	0.0654
Presence of mitosis	3/5 (60.0)	5/13 (38.5)	0.6078
Presence of hypercellularity	3/5 (60.0)	2/13 (15.4)	0.0987
Presence of well-formed blood vessels	3/5 (60.0)	1/13 (7.7)	0.0441
Abundant stroma	3/5 (60.0)	9/13 (69.2)	1.0000
Tumor necrosis	2/5 (40.0)	3/13 (23.1)	0.5827

## DISCUSSION

In this study, we examined the TFE3 and CAMTA1 protein expression profiles via immunohistochemistry and assessed the chromosomal alterations of *TFE3* and *CAMTA1* via FISH assays in EHEs. We also verified the presence of *YAP1-TFE3* and *WWTR1-CAMTA1* fusion transcripts by RT-PCR. These results indicate that these two chromosomal alterations were not mutually exclusive but compossible in EHEs. Therefore, we classified EHEs into two subgroups: TFE3-positive and TFE3-negative EHEs, and compared the clinical and histologic features of each. Notably, TFE3-positive EHEs seemed to present as a single larger mass compared with TFE3-negative EHEs. Similar to a previous report [6], we also observed an earlier onset of disease in patients with TFE3-positive EHEs than in patients with TFE3-negative EHEs and well-formed vascular channels in TFE3-positive EHEs on histological examination. In addition, we observed a tendency for TFE3-positive EHEs to more frequently demonstrate high-grade nuclear atypia and hypercellularity than TFE3-negative EHEs.

A previous study examined CAMTA1 expression profiles in EHEs and identified strong nuclear expression in most cases [8]. Consistent with these findings, we also demonstrated strong nuclear expression of CAMTA1 in 100.0% of EHEs. In the current study, we were able to identify TFE3 nuclear expression in five out of 18 cases (27. 8%). This ratio is much lower compared with that of a previous article which claimed to have observed TFE3 nuclear reaction in as much as 87.5% (*n* = 21/24) in their EHEs study population [7]. Our data also differs in that they reported *TFE3* rearrangement to occur in merely two cases (5.7%, *n* = 2/35) [7], whereas we detected YAP1-TFE3 gene fusion in 22.2% (*n* = 4/18) of EHEs and 80.0% (*n* = 4/5) of TFE3-positive EHEs based on FISH and RT-PCR results. EHE remains an extremely rare disease entity of which TFE3 expression has been scarcely examined. Although we add our current observations to the limited literature covering this subject, we acknowledge that subsequent studies which cover more cases are still required to further clarify such discrepancies, especially on TFE3 expression profile and YAP1-TFE3 gene fusion status that exist between our data and the previous report [7].

WWTR1 and YAP1 share conserved amino acid sequences for the WW domain that can interact with PDZ domains. They are named for the shared homology observed in postsynaptic density protein 95 (PSD95), *Drosophila* discs large tumor suppressor (Dlg1), and zonula occludens-1 protein (zo-1) [9]. Recently, a study described the mechanism of action of a WWTR1(TAZ)-CAMTA1 fusion oncoprotein, and the results suggested that the WWTR1-CAMTA1 fusion might cause resistance to anoikis and the oncogenic transformation of tumor cells [10]. This study implies that the WWTR1(TAZ)-CAMTA1 fusion oncoprotein might have a profound role in the molecular pathogenesis of EHEs. Despite the careful analyses reported in previous studies [6, 7, 11], questions still remain regarding how two very similar molecules could have different translocation partners in one specific tumor type. This concept will be examined in more detail in future studies.

In this study, we observed that TFE3-positive EHEs had a tendency to present as a single larger mass in younger patients compared with TFE3-negative EHEs and demonstrated histologic findings of the presence of high-grade nuclear atypia and hypercellular areas. We believe that these clinicopathologic findings in EHEs with both *WWTR1-CAMTA1* and *YAP1-TFE3* gene translocations, which were not identified in EHEs having only the *WWTR1-CAMTA1* translocation, might reflect a higher degree of genetic heterogeneity in these tumors. Traditionally, sarcomas have been classified into two broad categories: tumors with simple genetic alterations and tumors with complex and unbalanced karyotypes [12]. Tumors with complex genetic alterations are typified by genome instability, resulting in multiple genomic aberrations in the genome of a single tumor and the heterogeneity of aberrations across tumors of a given type [13, 14]. Although two distinct chromosomal alterations specific to EHEs have been identified, we presume that these two genetic alterations can occur simultaneously in EHEs, possibly reflecting an increased degree of genetic instability.

According to a previous study, tumor size was associated with decreased survival of EHE patients and tumors with >3 mitotic figures/50 high power fields and size >3.0 cm had the worst prognosis [2]. Interestingly, we observed that TFE3-positive EHEs presented as single masses with significantly larger sizes compared to TFE3-negative EHEs, which typically contained multiple lesions of smaller sizes. These data, together with the above findings, have newly established two clinically distinct subgroups of EHEs: TFE3-positive and TFE3-negative EHEs, which can be used to more accurately diagnose and treat EHEs in the future.

## MATERIALS AND METHODS

### Cases

This retrospective study was approved by the Institutional Review Board of Yonsei University Medical Center (approval number: 4-2014-0852). Our inclusion criteria defined a study population of 18 EHE patients who had been histologically diagnosed between 1993-2013. Archival tissues stained with hematoxylin and eosin (H&E) were reviewed by three pathologists (SK Kim, SJ Lee, and WI Yang).

### Immunohistochemistry

Tumors were fixed in formalin and embedded in paraffin. Briefly, 5 μm-thick sections were cut using a microtome, transferred onto adhesive slides, and dried at 62°C for 30 min. Immunohistochemistry with antibodies against CD31 (M0823, Dako, CA, USA), CD34 (M7165, Dako, CA, USA), TFE3 (MRQ-37, CA, USA), and CAMTA1 (ab64119, Abcam, Cambridge, UK) was performed using an automated immunohistochemical staining instrument (Ventana Discovery^®^ XT, Ventana Medical System, AZ, USA).

### Fluorescent *in situ* hybridization (FISH) assay

FISH was performed on tumor sections after examination by H&E microscopy. A ZytoLight SPEC TFE3 Dual Color Break-Apart Probe (Z-2109-200, ZytoVision GmbH, Bremerhaven, Germany) and a MacProbe^TM^ CAMTA1 Dual Color Break-Apart Probe (KH01M2NA12 and KH02M2NA12, Macrogen, South Korea) were used according to the manufacturer's instructions for the detection of translocations involving the *TFE3* gene at Xp11.23 and the *CAMTA1* gene at 1p36.23, respectively. Break-apart on the slides was evaluated using an epifluorescence microscope (Olympus, Tokyo, Japan). During interphase in normal cells without translocations, green/orange fusion signals appear. Xp11.23 or 1p36.23 loci affected by translocations are indicated by separate and distinct green and orange signals. A case was interpreted as positive when at least 10 out of 50 counted tumor cells (20%) showed separated green and orange signals [7].

### Reverse transcription polymerase chain reaction (RT-PCR)

Isolation of RNA from formalin-fixed, paraffin-embedded (FFPE) samples was performed following the manufacturer's protocol (RNeasy FFPE kit, Qiagen, Valencia, CA). RT-PCR was performed as previously published [3, 6, 7] using the following primers to identify the *WWTR1-CAMTA1* fusion transcript or the *YAP1-TFE3* fusion transcript: *WWTR1* exon 4 forward, 5′-CCGTCAGTTCCACACCAGTGCCTC-3′; CAMTA1 exon 9 reverse, 5′-GGGGCTACAGCAGGGGAGGC-3′; *YAP1* exon 1 forward, 5′-CCTGGAGGCGCTCTTGAACG-3′; *TFE3* exon 6 reverse, 5′-GTTGCTGACAGTGATGGCTGG3′. The RT-PCR products were analyzed by electrophoresis.

### Statistics

All statistical analyses were performed using GraphPad Prism 5 software, version 5.01 (GraphPad Software Inc., La Jolla, CA, USA) and SPSS for Windows, version 20.0 (SPSS Inc., Chicago, IL, USA). For the analysis of age at diagnosis and tumor size, a significant difference between means was determined by t-tests. Sex, tumor multiplicity, and metastasis were compared by Chi-square analyses and Fisher's exact tests.

## SUPPLEMENTARY MATERIAL DATA


