# Supplementary material for: Epithelioid hemangioendotheliomas with TFE3 gene translocations are compossible with CAMTA1 gene rearrangements
Source: Oncotarget. 2016 Jan 28;7(7):7480–8. doi: 10.18632/oncotarget.7060 (PMC4884933; doi:10.18632/oncotarget.7060)
Supplement: Supplementary file 1 [file oncotarget-07-7480-s001.pdf]

Epithelioid hemangioendotheliomas with *TFE3* gene translocations are compossible with *CAMTA1* gene rearrangements

Supplementary Material

Supplementary Data 1. Histologic findings of 18 EHEs

Case	Histologic features							
	Cellular feature	Cytoplasmic feature	Nuclear atypia	Amount of stroma	Hypercellular area	Blood vessel formation	Mitosis (/10HPFs)	Tumor necrosis
1	Spindle and epithelioid	Foamy	Mild	Moderate	Not identified	Moderate	0	-
2	Epithelioid	Eosinophilic	Mild	Moderate	Not identified	Moderate	0	-
3	Epithelioid	Eosinophilic	Moderate	Abundant	Not identified	Moderate	0	-
4	Epithelioid	Eosinophilic	Mild	Abundant	Not identified	Moderate	0	-
5	Epithelioid	Eosinophilic	Mild	Abundant	Not identified	Moderate	0	-
6	Spindle and epithelioid	Foamy	Mild	Moderate	Present	Well	2	-
7	Spindle and epithelioid	Eosinophilic	Moderate to focal high grade	Abundant	Present	Well	0	-
8	Epithelioid	Eosinophilic	Moderate	Moderate	Present	Well	1	-
9	Epithelioid	Eosinophilic	Moderate	Abundant	Not identified	Moderate	1	+
10	Epithelioid	Foamy	Mild	Abundant	Not identified	Moderate	0	+
11	Epithelioid	Eosinophilic	Moderate	Abundant	Not identified	Moderate	0	-
12	Spindle and epithelioid	Eosinophilic	Mild	Abundant	Not identified	Moderate	1	-
13	Epithelioid	Eosinophilic	Moderate	Abundant	Not identified	Moderate	1	+

14	Epithelioid	Eosinophilic	Moderate	Abundant	Not identified	Moderate	1	+
15	Spindle and epithelioid	Foamy	Mild	Abundant	Not identified	Moderate	0	-
16	Spindle and epithelioid	Foamy	Moderate	Moderate	Present	Moderate	1	-
17	Epithelioid	Foamy	Moderate to focal high grade	Moderate	Present	Moderate	1	+
18	Epithelioid	Eosinophilic	Moderate	Abundant	Not identified	Moderate	0	-

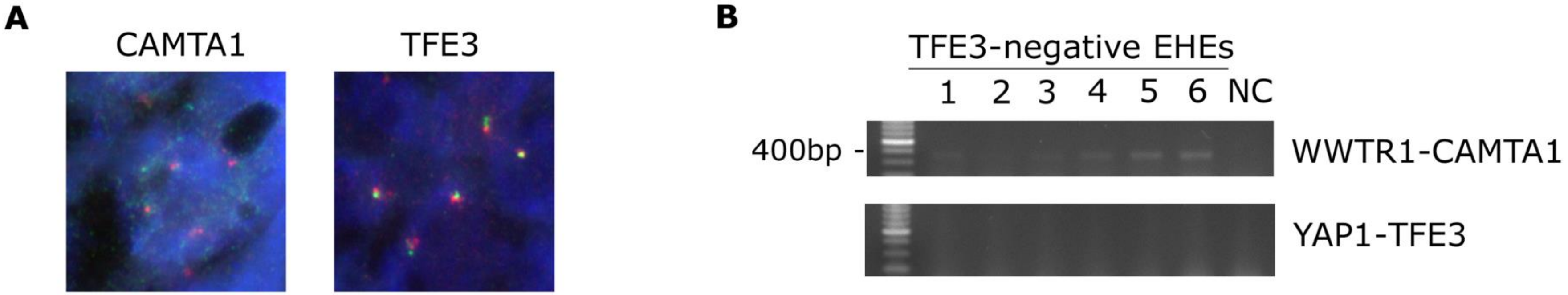

Supplementary Data 2. CAMTA1 translocation in EHEs without TFE3 rearrangements.
A. FISH assay results for TFE3 and CAMTA1 break-apart showing separated green and orange signals only within the CAMTA1 gene but not the TFE3 gene (1,000× magnification). B. RT-PCR assay results identifying the presence of fusion transcripts of WWTR1-CAMTA1 genes but not YAP1-TFE3 genes. N/C, negative control.

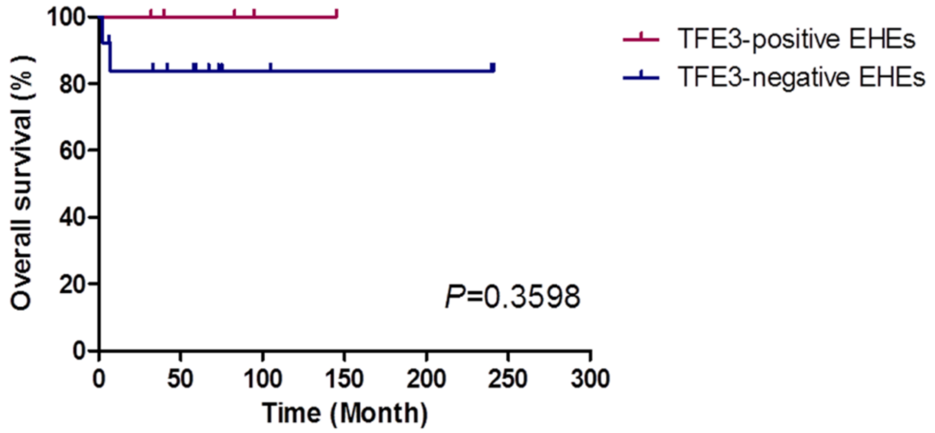

Supplementary Data 3. Comparison of the overall survival between patients with TFE3-positive and TFE3-negative EHEs by Kaplan-Meier analysis
